# Early career interview: Marjorie Pizarro-Guajardo, Universidad Andrés Bello

**DOI:** 10.2144/fsoa-2019-0146

**Published:** 2020-01-29

**Authors:** Marjorie Pizarro-Guajardo

**Affiliations:** 1 Andrés Bello National University, Santiago, Chile

**Keywords:** *Clostridium difficile*, infection, spores, treatment

## Abstract

Marjorie Pizarro-Guajardo is a postdoctoral fellow at the Universidad Andrés Bello (Santiago, Chile), where she studies *Clostridium difficile* spores. She won the 2019 Future Science Future Star Award. Here she tells us about her career to date, and how she felt winning the award.

## Please tell us about yourself

I studied molecular biotechnology as an undergraduate at the University of Chile (Santiago, Chile), inspired by an interest that my high school biology professor, Nelly Parra, helped me to find. In 2011, I started to study the anaerobic pathogen *Clostridium difficile*, the pathogen responsible for nosocomial diarrhea, and how the spores contribute to the infection process. I felt a high level of passion for my work and after 1 year as Research Assistant I was accepted in the PhD program of Biotechnology at tge University Andrés Bello (Santiago, Chile), where I focused my work on the development of a strategy to remove *C. difficile* spores from the host to make a therapy that can improve the clearance of the infection.

## What made you choose a career in your field?

I chose microbiology because I am interested in the complex interactions of bacteria that shape the environment where they live. My first introduction to microbiology was ecological microbiology during my undergraduate degree. Later I moved to the field of pathogenic microbes, looking to learn about interactions between bacteria and its environment. It was very exciting to start to work with a microorganism that requires biosafety level II work practices to be set, in a new laboratory installed by Dr Daniel Paredes-Sabja in Chile.

## What are the main highlights of your career so far?

When we found that the removal of spores from the colonic environment can reduce the *C. difficile* infection and the recurrence, we continued working on the development of an improved treatment. In order to develop this treatment, we characterized the immunoreactive proteins in the spore. These two highlights were the basis for a grant application that gave us funding for 2 years of postdoc work and for the development of the project.

## What is the most difficult challenge you have encountered in your work & how did you overcome it?

The biggest difficulty faced in my work is reagent acquisition; the delivery from Europe and USA commonly takes 60 days for specific reagents like antibodies, special protein purification kits or restriction enzymes, due to customs issues. We have a similar problem with services like DNA synthesis, mass spectrometry analysis and DNA sequencing, things that cannot be performed in Chile with good quality results, and we need to request the analysis in other countries. For frequently used reagents, the programmed buying of reagents can help us to overcome this problem, but when a good experimental idea requires a special reagent, we must wait.

As we cannot afford to lose such valuable time, the way to overcome this is to use this waiting time on side projects that contribute partially to the main goal.

## What is your favorite publication so far?

The best publication I have is the ‘Characterization of chicken IgY specific to clostridium difficile R20291 spores and the effect of oral administration in mouse models of initiation and recurrent disease’, published in *Frontiers in Cellular and Infection Microbiology* [1]. I worked with two undergrad students for 2 years to evaluate the effect of an anti-spore passive immunization, the result of which is a delay in the development of diarrhea symptoms, indicating that removal of spores from the host can improve the resolution of the disease. This work is the starting point for my current work focused on the optimization of the treatment and the characterization of external-layer structures in *C. difficile* spore.

## What are your main aims for the future?

My major aim for the future is to continue with my training and be able to contribute to scientific research excellence by tackling big research questions and developing my own research path and independence. In this sense, I envision myself developing novel therapies that will aid in the prevention and treatment of bacterial infections. In this context, I expect to specifically demonstrate that spore-surface proteins are outstanding candidates for vaccine development. As a next stage, I would like to take my research further and develop a vaccination prototype and scale this to clinical trails through NIH-based funding schemes. Unlike most scientists, my plans are to create this research independence environment within my current research group, as I plan to contribute to its growth and future development.

## Where do you hope to see yourself in 5 years?

In 5 years, I see myself leading my research group and addressing the relevant questions that appear in the future related to *C. difficile* infection and therapeutic development. Despite the fact that doing science in Chile is already difficult due to the tremendous amount of hurdles that we have to surpass, (i.e., bureaucracy, high reagent prices, delay in reagent delivery, low science funding schemes) I have faced big scientific questions, and I believe that I can figure out how to use first-world technology to keep addressing important goals in my current environment in Chile, contributing to the development of science in my country.

My PI has given me a tremendous amount of freedom to develop my research independence and skills. I truly believe that this will end in a synergistic group where we will be able to tackle high caliber research questions in the future.

## How do you feel about winning?

I am really happy because this gives me the opportunity to show all of my research and my passion about this work to other people, not just fellow scientists but to my family and friends and friends of my friends as well. That was very interesting because I had many people from my social network ask me about my work which is great because it means we are moving the science out of the lab and into society.

## What are your top three tips for other early career researchers aspiring to emulate your success?

The first would be, try to be visible – try to take your research off of your benchtop and talk about what you do in normal words to other people in society because, ultimately, all that we do has to have a repercussion in society. So that would be the first. The second would be to go networking. Contact with other scientists in other parts of the world can help us to improve our own research, resulting in more high-quality papers and research. The third would be never give up. A science career is hard – we all know that – but what makes us go through it no matter what is the passion that we feel for it, so never stop feeling this passion and never give up.
